# Effects of 1-day outpatient nutrition intervention on insulin resistance and maternal–infant outcomes in women with gestational diabetes mellitus: a single-center retrospective cohort study

**DOI:** 10.3389/fnut.2026.1792550

**Published:** 2026-07-22

**Authors:** Yan Feng, Yongchao Zhang, Xiaowei Li, Qi Feng, Yinghong Zhang

**Affiliations:** 1 Department of Clinical Nutrition, Yuhuangding Hospital affiliated to Qingdao University, Yantai, China; 2 Department of Clinical Nutrition, Weifang Traditional Chinese Medicine Hospital, Weifang, China; 3 Military Burn Center, The 990th Hospital of The People’s Liberation Army, Zhumadian, China; 4 Department of Obstetrics, Yuhuangding Hospital affiliated to Qingdao University, Yantai, China

**Keywords:** GDM, insulin resistance, iron, one-day outpatient, stress adaptation

## Abstract

**Aim:**

This study aimed to investigate the effect of a 1-day outpatient intervention on insulin resistance in women with gestational diabetes mellitus (GDM), to explore its potential mechanisms, and to evaluate its impact on maternal and neonatal outcomes so as to optimize clinical intervention strategies and provide evidence for ensuring maternal and neonatal safety.

**Methods:**

A total of 252 patients with GDM were included in this study, and they were divided into the GDM intervention group (receiving 1-day outpatient comprehensive management mode of diabetes) and the GDM group (routine nutrition consultation). All participants were followed up to 6-week postpartum. Variance analysis, chi-square test, and bivariate Pearson’s correlation were used to analyze changes in blood glucose, stress hormones, iron metabolism, and oxidative stress markers, as well as their correlations.

**Results:**

After intervention, fasting insulin, HOMA-IR, and 2-h postprandial blood glucose were significantly decreased in the GDM intervention group (all *p* < 0.05). Levels of epinephrine (E), cortisol, MDA, and serum ferritin (SF) were also lower than in the GDM group (all p < 0.05). The rates of insulin use, postpartum glucose abnormality, preterm birth, and macrosomia were significantly reduced (all *p* < 0.05). E, cortisol, MDA, and SF were positively correlated with HOMA-IR (*r* = 0.457, 0.532, 0.324, 0.321, all *p* < 0.05).

**Conclusion:**

One-day outpatient intervention can effectively alleviate insulin resistance, lower postprandial blood glucose, and improve maternal and infant outcomes in GDM patients. The mechanism may be associated with improved iron metabolism, reduced stress disorder, and oxidative stress injury in GDM patients.

## Introduction

As one of the common metabolic complications during pregnancy, gestational diabetes mellitus (GDM) has become a major concern in the field of perinatal medicine worldwide. GDM not only significantly increases the risk of preterm delivery, preeclampsia, cesarean section, and other adverse pregnancy outcomes but also may lead to abnormal fetal growth and development, such as small for gestational age (SGA), large for gestational age (LGA), and macrosomia, while increasing the incidence of postpartum type 2 diabetes in mothers (1–3). In recent years, with changes in lifestyle and improved coverage of pregnancy screening, the incidence rate of GDM has shown a significant upward trend. The incidence rate of GDM in China has reached 11.7% (4), making it an important public health concern affecting the health of mothers and infants.

Although adverse effects of GDM has been widely recognized and clinical attention to the condition continues to increase, the current prevention strategies for GDM still lack broad applicability, and treatment measures are mostly focused on blood glucose monitoring and basic intervention, without establishing a precise and effective comprehensive management system (5–7). Our previous 1-day outpatient intervention study on diabetes preliminarily demonstrated that this intervention model can effectively reduce the stress adaptation disorder of GDM patients and improve blood glucose-related indicators. However, the mechanism of improvement remains unclear (8). In addition, insufficient evidence exists to determine whether the abnormal iron metabolism of GDM patients plays a role in it and whether this intervention can further reduce the risk of adverse maternal and fetal outcomes. Based on the above research gaps, this study aims to deeply explore the potential molecular mechanism of the 1-day outpatient intervention for diabetes to improve insulin resistance in GDM patients and to systematically evaluate its impact on maternal and fetal outcomes so as to provide a new basis for optimizing the clinical intervention strategy for GDM and improving maternal and infant health.

## Research design and methods

### Subjects

Pregnant women who underwent prenatal checkups at Yantai Yuhuangding Hospital from April 2023 to August 2025 and were between 24 and 28 weeks pregnant. These pregnant women fasted for 8–10 h, rested for 15–30 min after admission in the morning, had fasting venous blood collected, and then underwent a 75-g oral glucose tolerance test. The diagnostic criteria for gestational diabetes were based on the International Association of Diabetes and Pregnancy Study Groups (IADPSG) and World Health Organization (WHO) standards: At 24–28 weeks of pregnancy, fasting blood glucose ≥92 mg/dL (5.1 mmol/L), 1-h glucose ≥180 mg/dL (10.0 mmol/L), or 2-h glucose ≥153 mg/dL (8.5 mmol/L) are the standard values, and if they exceed this standard, they can be diagnosed as GDM (9).

**Figure 1:**
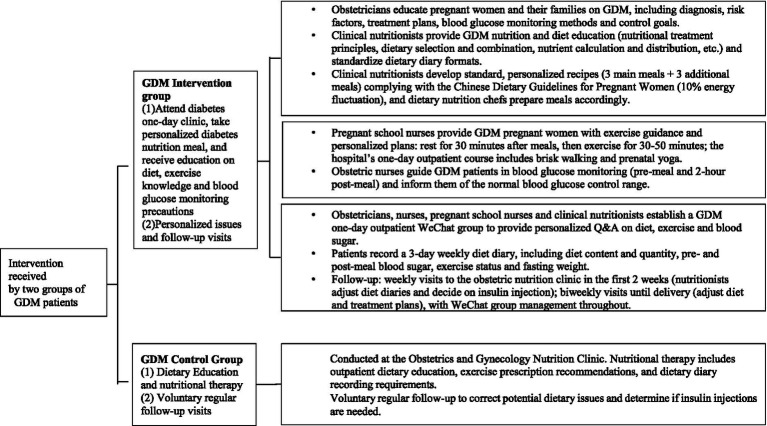
One-day outpatient nursing management intervention model.

We included 252 patients with GDM. Patients diagnosed with GDM were divided into two groups. One group was the GDM intervention group, which voluntarily participated in the 1-day outpatient service for diabetes (at their own expense), based on the comprehensive management model of the 1-day outpatient service for diabetes (Figure 1). The other group was the GDM group, which received regular nutritional counseling. Both groups are regularly followed up.

The members of the diabetes 1-day clinic include obstetricians, clinical nutritionists, nurses, patients in the GDM intervention group, and their families. The diabetes 1-day clinic is held at the pregnant women’s school, and health education tools, food models, blood glucose detectors, and yoga mats, among other tools, are prepared. The course is held once a month, with 8–10 people per session.

### One-day outpatient nursing intervention model for gestational diabetes

1. Obstetricians teach patients and their families, including disease diagnosis, high-risk factors, specific treatment plans, blood glucose monitoring guidance methods, and blood glucose control goals;
2. Clinical nutritionists carry out nutrition treatment knowledge education for gestational diabetes, including nutrition treatment, diet selection, diet matching, calculation and distribution of daily nutrition requirements, nutrition communication guidance, etc., and guide GDM patients to record a diet diary;
3. Clinical nutritionists develop personalized recipes, including 3 nutritious meals and 3 additional meals, and professional nutrition chefs prepare meals based on the recipes. The energy intake of the whole-day diet conforms to the 2023 edition of the Chinese Dietary Reference Intake for Nutrients, with an initial energy of 1700 kcal; it increases by 250 kcal and 400 kcal in the middle and late stages of pregnancy, respectively, with an energy fluctuation of 10%.
4. The obstetric nurses provide exercise guidance for GDM pregnant women and develop personalized exercise plans. On the day of outpatient services for diabetes, they take a 30-min rest after lunch and start exercising for 30–50 min, including hospital walking and yoga for pregnant women, simultaneously guiding GDM patients in blood glucose monitoring.
5. All personnel provide on-site personalized Q&A for GDM patients; establish a GDM daily outpatient WeChat group to answer various questions about daily diet and blood glucose status for GDM patients;
6. The patient recorded a daily diet diary for 3 days a week, including the content and quantity of the diet, blood glucose levels before and after each meal, exercise volume, and weight measurement on an empty stomach in the morning. During the first 2 weeks, regular follow-up visits will be conducted at the obstetrics nutrition clinic each week. The clinical nutritionist corrects any issues in the patient’s diet diary and decides whether to inject insulin. Throughout the follow-up period, GDM patients were managed in a dedicated WeChat group. Blood glucose was monitored, and personalized inquiries were addressed. Regular follow-up visits were arranged every 3–4 weeks through 42 days postpartum.

#### Inclusion and exclusion criteria

Inclusion criteria:

1. Age between 20 and 40 years old;
2. Singleton pregnancy;
3. Pre-pregnancy fasting blood glucose (FPG) <6.1 mmol/L.

Exclusion criteria:

1. Pregnant women with a history of type 1 diabetes, type 2 diabetes, hypothyroidism, hyperthyroidism, and other diseases in the past;
2. Pregnant women with a history of using hormones or other medications that affect blood glucose levels;
3. Pregnant women with mental or expressive disorders;
4. Pregnant women with autoimmune diseases or infectious diseases;

We obtained approval from the Ethics Committee of Yantai Yuhuangding Hospital [YanYu Ethics Review (2022-264)] and obtained informed consent from all participants and registered with the Chinese Clinical Trial Registry (ChiCTR2300070351).

### Characteristics of GDM patients

**Figure 2:**
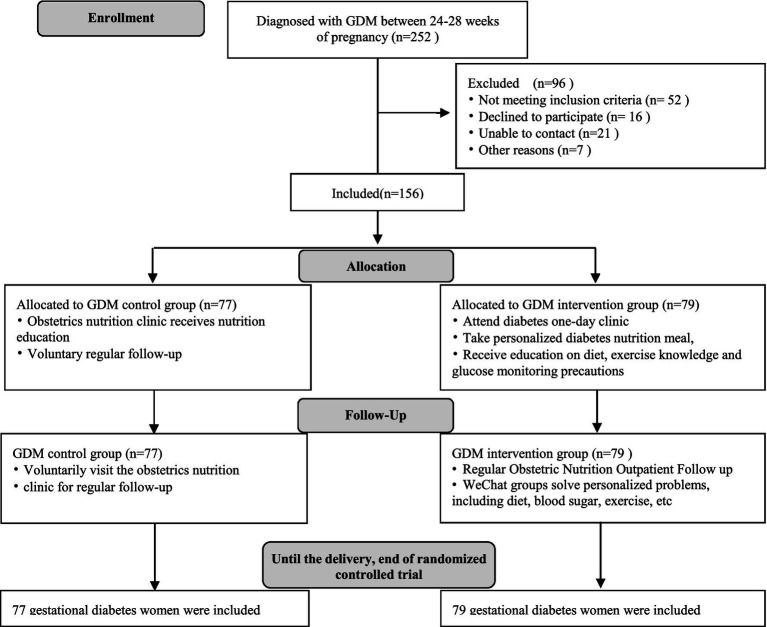
Flowchart of enrollment and allocation of GDM women.

This study included 252 pregnant women diagnosed with GDM between 24 and 28 weeks and 52 patients who did not meet the inclusion and exclusion criteria, declined to participate (*n* = 16), were unable to be contacted (*n* = 21), or for other reasons (*n* = 7). There were 79 participants in the GDM intervention group and 77 participants in the GDM group (Figure 2).

The clinical characteristics of GDM patients include age, pre-pregnancy body mass index (BMI), gestational weeks, and pre-inclusion weight gain. Pre-pregnancy BMI was calculated as pre-pregnancy weight (kg)/height (m^2^).

### Blood glucose, stress hormones, oxidative stress markers, and iron metabolism-related indicators

All enrolled patients had fasting venous blood, 1-h venous blood, and 2-h venous blood samples collected. Fasting venous blood was used to detect fasting blood glucose, insulin, glycated hemoglobin (HbA1c), stress hormones, oxidative stress markers, and iron metabolism-related indicators. One-h and 2-h serum samples were primarily used to measure 1-h and 2-h blood glucose levels.

The glucose oxidase method was used to measure blood glucose, while electrochemiluminescence immunoassay (Roche Diagnostics, Mannheim, Germany) was used to detect fasting insulin levels and cortisol. Radioimmunoassay was used to measure adrenaline (E) and noradrenaline (NE) levels, and hemoglobin (Hb) was measured using a fully automated Roche biochemical analyzer (Roche Diagnostics, Mannheim, Germany). All reagents were Roche-matched reagents. The homeostatic model for the assessment of insulin resistance (HOMA-IR) was used to evaluate the degree of insulin resistance (IR). HOMA-IR = Fins(mU/L)*FPG(mmol/L)/22.5 (10). Malondialdehyde (MDA), superoxide dismutase (SOD), and reduced glutathione (GSH) were used as markers of oxidative stress damage and were detected using corresponding assay kits (Nanjing Institute of Bioengineering, China). The results were expressed in μmol/L, U/mL, and mg/L, respectively; ELISA was used to detect serum iron (Fe) levels, while immunoturbidimetry was used to measure total iron-binding capacity (TIBC) and serum ferritin (SF) concentrations. Transferrin saturation (TS) was calculated as Fe/TIBC *100%.

### Maternal and neonatal outcomes

Under the comprehensive management model, both groups of patients were followed-up until 6 weeks postpartum. Outcomes of GDM patients: whether insulin injection, cesarean sec-tion, gestational hypertension, preeclampsia, premature rupture of membranes, preterm birth, etc.

After delivery, GDM patients underwent OGTT again at 6 weeks postpartum. If fasting blood glucose (FPG) was <100 mg/dL (5.6 mmol/L) and 2-h blood glucose was <140 mg/dL (7.8 mmol/L) at 6 weeks postpartum, glucose metabolism was considered normal. A fasting blood glucose of 5.6–6.9 mmoL/L (100–126 mg/dL) was defined as impaired fasting blood glucose (IFG), whereas a 2-h blood glucose concentration of 7.8–11.0 mmoL/L was classified as impaired glucose tolerance (IGT). Simultaneously, gestational weight gain was monitored among GDM patients until childbirth.

Infant outcomes: Count for macrosomia (birth weight>4,000 g), large for gestational age (LGA), small for gestational age (SGA), NICU neonatal monitoring, perinatal mortality rate, incidence of hypoglycemia, and recorded fetal birth weight.

### Statistical analysis

SPSS 21.0 statistical analysis software (SPSS, Inc., Chicago, Illinois, USA) was used to manage the database and analyze the data. The data were expressed as mean ± standard error. After passing the normality test and homogeneity of variance tests, one-way ANOVA was used to compare the means between two groups. After logarithmic transformation, data with a non-normal distribution were compared. We used a chi-square test to compare maternal and infant outcomes between two groups, including indicators related to maternal and neonatal outcomes.

We conducted normality tests on all continuous variables and used Pearson’s correlation analysis to evaluate the correlation between stress hormones, oxidative stress markers, and HOMA-IR, as well as the correlation between oxidative stress markers and iron metabolism-related indicators, for variables that followed a normal distribution. All hypothesis tests were performed on both sides. A *p*-value of <0.05 was used as the statistical significance criterion.

## Results

### Maternal characteristics

The maternal characteristics, including maternal age, gestational weeks, pre-gestational BMI, and pregnancy weight gain at initial inclusion, were similar between the GDM group and the GDM intervention group at the start of the study; there was no statistically significant difference between the two groups (*p* > 0.05) (Table 1).

**Table 1:** Maternal characteristics in GDM women (*x̄* ± *s*).

Maternal characteristics	GDM group (*n* = 77)	GDM intervention group (*n* = 79)	*F*	*p*
Maternal age (years)	32.31 ± 4.12	32.70 ± 5.13	0.004	0.987
Gestational weeks (weeks)	27.03 ± 2.28	28.30 ± 3.43	1.001	0.318
Pre-gestational BMI (Kg/m^2^)	25.04 ± 4.93	24.88 ± 3.82	0.098	0.755
Gestational weight gain pre-enrollment (kg)	9.18 ± 4.80	9.48 ± 5.19	0.279	0.598

BMI, the body-mass index is the weight in kilograms divided by the square of the height in meters.

### Blood glucose, stress hormones, oxidative stress, and iron metabolism-related indicators after intervention in two groups

#### Blood glucose

Compared with the GDM group, the postprandial 2-h blood glucose of patients in the GDM intervention group decreased by 5.60% (*p* < 0.05). After intervention, fasting insulin and HOMA-IR levels also showed a downward trend, decreasing by 5.38 and 5.92%, respectively, in the GDM intervention group (both *p* < 0.05). There was no significant difference in fasting blood glucose and HbA1C between the two groups (both *p* > 0.05; Table 2). The blood glucose-related results suggest that after 1-day outpatient intervention, 2-h postprandial blood glucose and insulin resistance in GDM patients have improved to a certain extent.

**Table 2:** Blood glucose, stress hormones, oxidative stress, and iron metabolism-related indicators in GDM women (*x̄* ± *s*).

Indicators		GDM group	GDM intervention group	*F*	*p*
Blood glucose	Fasting blood glucose (mmol/L)	5.89 ± 0.93	5.94 ± 0.77	0.241	0.624
	Postprandial blood glucose at 2 h (mmol/L)	6.96 ± 1.63	6.57 ± 1.29^*^	5.333	0.022
	Fasting insulin	17.47 ± 3.55	16.53 ± 4.27^*^	4.484	0.035
	HOMA-IR	4.56 ± 2.16	4.29 ± 1.591^*^	4.126	0.043
	HbA1C (%)	5.61 ± 0.40	5.52 ± 0.40	1.725	0.190
Stress hormones	E (ng/L)	247.15 ± 94.11	219.72 ± 96.31^*^	6.409	0.012
	NE (ng/L)	171.42 ± 73.88	157.41 ± 80.28	2.931	0.088
	Cortisol (nmol/L)	306.32 ± 133.91	272.73 ± 93.88^**^	6.982	0.009
Oxidative stress	MDA (μmol/L)	9.73 ± 3.59	8.74 ± 4.15^*^	6.29	0.013
	SOD (U/mL)	159.56 ± 64.94	143.26 ± 36.72	7.48	0.007
	GSH (mg/L)	29.41 ± 11.72	30.12 ± 9.55	0.363	0.547
Iron metabolism	Fe (μmol/L)	10.57 ± 3.13	9.98 ± 2.71	3.151	0.077
	SF (μg/L)	264.90 ± 115.40	237.94 ± 96.80^*^	4.979	0.026
	TS	0.32 ± 0.15	0.29 ± 0.13	2.806	0.095
	Hb (g/L)	115.02 ± 12.72	113.34 ± 13.91	1.235	0.267

HOMA-IR, homeostasis model assessment for insulin resistance; E, epinephrine; NE, noradrenaline; MDA, malondialdehyde; SOD, superoxide dismutase; GSH, glutathione; SF, serum ferritin; TS, transferrin saturation; Hb, hemoglobin.

^*^Indicates *p* < 0.05 vs. GDM group.

^**^Indicates *p* < 0.01 vs. GDM group.

#### Stress hormones and oxidative stress

Levels of E and cortisol were decreased by 11.10 and 10.97%, respectively, in the GDM intervention group (*p* < 0.05 and *p* < 0.01). The MDA level was 10.17% and the SOD level was 10.22% lower than those in the GDM group (*p* < 0.05 and *p* < 0.01), and there was no statistically significant difference in GSH between the two groups (*p* > 0.05) (Table 2).

#### Iron metabolism

Compared with the GDM group, the SF level decreased by 10.18% in the GDM intervention group (*p* < 0.05), and Fe, TS, and Hb levels were 5.58, 9.38, and 1.46% lower in the GDM intervention group than those in the GDM group; however, the differences were not statistically significant (all *p* > 0.05) (Table 2).

### The incidence of maternal postpartum outcomes and fetal outcome indicators in the two groups

#### Maternal postpartum outcomes

The insulin injection utilization rate in the GDM intervention group [4/79 (5.1%)] was significantly lower than that in the GDM group [13/77 (16.9%)] (*p* < 0.01); postpartum blood glucose abnormality [4/79 (5.1%)] was also significantly lower than that in the GDM group [12/77 (15.6%)] (*p* < 0.01). Incidence of cesarean section, pregnancy-induced hypertension, preeclampsia, and premature rupture of membranes in the GDM intervention group was 5/79 (6.4%), 4/79 (5.1%), 3/79 (3.8%), and 2/79 (2.6%), respectively, which was lower than those in the GDM group; however, there was no statistically significant difference (all *p* > 0.05) (Table 3).

**Table 3:** Incidence of maternal postpartum and fetal outcome indicators in two groups.

Maternal postpartum and fetal outcome indicators	GDM group		GDM intervention group		*p*
	95%CI		95%CI		
Insulin injection	13/77 (16.9%)	9.4–26.8%	4/79 (5.1%)	1.4–12.7%	0.000
Cesarean section	6/77 (7.8%)	2.9–16.2%	5/79 (6.4%)	2.1–14.4%	0.661
Pregnancy-induced hypertension	6/77 (7.8%)	2.9–16.2%	4/79 (5.1%)	1.4–12.7%	0.502
Preeclampsia	5/77 (6.5%)	2.2–14.5%	3/79 (3.8%)	0.8–10.8%	0.283
Premature rupture of membranes	4/77 (5.2%)	1.4–12.8%	2/79 (2.6%)	0.3–9.0%	0.406
Postpartum blood glucose abnormality	12/77 (15.6%)	8.4–25.7%	4/79 (5.1%)	1.4–12.7%	0.001
Preterm birth	7/77 (9.1%)	3.7–17.8%	3/79 (3.8%)	0.8–10.8%	0.033
Macrosomia	18/77 (23.4%)	14.6–34.3%	5/79 (6.4%)	2.1–14.4%	0.000
LGA	12/77 (15.6%)	8.4–25.7%	7/79 (9.0%)	3.7–17.7%	0.166
SGA	5/77 (6.5%)	2.2–14.5%	6/79 (7.7%)	2.9–16.1%	0.510
NICU neonatal monitoring	5/77 (6.5%)	2.2–14.5%	4/79 (5.1%)	1.4–12.7%	0.192
Perinatal death	1/77 (1.3%)	0.0–7.0%	1/79 (1.3%)	0.0–6.9%	0.617
Incidence of neonatal hypoglycemia	5/77 (6.5%)	2.2–14.5%	4/79 (5.1%)	1.4–12.7%	0.312

LGA, large for gestational age; SGA, small for gestational age. ^*^Indicates *p* < 0.05 vs. GDM group. ^**^Indicate *p* < 0.01 vs. GDM group.

#### Fetal outcomes

The incidences of preterm birth and macrosomia were lower in the GDM group than in the GDM intervention group, with rates of 7/77 (9.1%) and 3/79 (3.8%), respectively, and both incidences were lower in the GDM intervention group than those in the GDM group (*p* < 0.05, <0.01). LGA, SGA, NICU neonatal monitoring, perinatal death, and incidence of neonatal hypoglycemia in the GDM intervention group were 7/79 (9.0%), 6/79 (7.7%), 4/79 (5.1%), 1/79 (1.3%), and 4/79 (5.1%), respectively; there were no statistically significant differences (all *p* > 0.05) (Table 3).

### Maternal and fetal weight and postpartum blood glucose-related indicators

Weight gain of pregnant women in the GDM intervention group was significantly lower than that in the GDM group (*p* < 0.01); both gestational week at delivery and postpartum 2-h blood glucose were significantly decreased (*p* < 0.01, <0.05). There was no statistically significant difference in body mass index at delivery, postpartum fasting blood glucose, and fetal birth weight between the two groups (all *p* > 0.05) (Table 4).

**Table 4:** Maternal and fetal weight and postpartum blood glucose-related indicators.

Weight and postpartum blood glucose related indicators	GDM group	GDM intervention group	*F*	*p*
Weight gain (at the time of production)	5.06 ± 2.02	3.01 ± 1.28^**^	19.22	0.000
Body-mass index at delivery (kg/m^2^)	30.36 ± 4.78	29.61 ± 3.85	2.36	0.126
Delivery gestational week	37.13 ± 1.72	37.98 ± 1.62^**^	8.495	0.004
Postpartum fasting blood glucose	4.64 ± 0.74	4.69 ± 0.82	0.421	0.517
Postpartum 2-h blood glucose	7.48 ± 1.53	7.18 ± 0.77^*^	5.11	0.024
Fetal birth weight	3374.69 ± 496.93	3293.75 ± 463.77	2.200	0.139

^*^Indicate *p* < 0.05.

^**^Indicate *p* < 0.01.

### Correlation of oxidative stress and iron metabolism indicators with HOMA-IR in two groups

In the GDM intervention group, E, cortisol, MDA, and SF were positively correlated with HOMA-IR (*r* = 0.457, 0.532, 0.324, 0.321, *p* < 0.05, 0.01, 0.01, 0.01), but no significant correlation between BMI, weight gain, HbA1C, NE, SOD, GSH, Fe, TS, Hb, and HOMA-IR was observed (all *p* > 0.05) (Table 5). In the GDM group, only cortisol was positively correlated with HOMA-IR (*r* = 0.423, *p* < 0.01).

**Table 5:** Correlation of oxidative stress and iron metabolism indicators with HOMA-IR.

Groups			BMI	Weight gain	HbA1C	Stress hormones			Oxidative stress			Iron metabolism-related indicators			
						E	NE	Cortisol	MDA	SOD	GSH	Fe	SF	TS	Hb
GDM intervention group	HOMA-IR	*r*	−0.167	0.039	0.047	0.457^*^	0.527	0.532^**^	0.324^**^	−0.157	0.261	0.062	0.321^**^	0.082	−0.172^**^
		*p*	0.534	0.422	0.365	0.033	0.122	0.002	0.000	0.212	0.087	0.633	0.000	0.334	0.227
GDM group	HOMA-IR	*r*	0.115	0.219	0.122	0.389	0.266	0.423^**^	0.237	−0.143	0.321	0.044	0.233	0.113	0.232
		*p*	0.643	0.453	0.237	0.073	0.154	0.001	0.119	0.211	0.145	0.412	0.092	0.523	0.311

^*^Indicate *p* < 0.05.

^**^Indicate *p* < 0.01.

### Correlation of oxidative stress with iron metabolism indicators in two groups

In the GDM intervention group, E, cortisol, Fe, SF, and HOMA-IR were positively correlated with MDA (*r* = 0.512, 0.449, 0.352, 0.317, 0.324, *p* < 0.01, <0.01, <0.05, <0.05, <0.01), while other hormone-related indicators were not significantly correlated with MDA (*p* > 0.05); HbA1C, E, cortisol, Fe, and TS were negatively correlated with SOD (*r* = −0.154, −0.357, −0.211, −0.244, −0.384, *p* < 0.05, <0.01, <0.05, <0.01, <0.01). There were no correlations observed between SOD and other indicators (all *p* > 0.05) (Table 6).

**Table 6:** Correlation between oxidative stress and iron metabolism indicators.

Oxidative stress indicators		BMI	Weight gain	HbA1C	Stress hormones			Iron metabolism-related indicators					HOMA-IR
					E	NE	Cortisol	Fe	SF	TS	Hb		
MDA	*r*	−0.144	0.135	0.089	0.512^**^	0.332	0.449^**^	0.352^*^	0.317^*^	0.343	−0.188		0.324^**^
	*p*	0.156	0.213	0.543	0.003	0.261	0.000	0.022	0.011	0.301	0.086		0.000
SOD	*r*	0.009	0.080	−0.154^*^	−0.357^**^	0.522	−0.211^*^	−0.244^**^	0.434	−0.384^**^	0.265		−0.157
	*p*	0.906	0.293	0.043	0.004	0.231	0.028	0.001	0.132	0.000	0.528		0.212
GSH	*r*	0.090	0.092	−0.077	−0.216	0.581^**^	0.341	0.189	0.622^**^	0.073	0.192		0.261
	*p*	0.241	0.231	0.314	0.132	0.000	0.122	0.572	0.000	0.389	0.066		0.087

^*^Indicate *p* < 0.05.

^**^Indicate *p* < 0.01.

## Discussion

Previous studies have shown that pregnant women are in a long-term chronic stress state, while GDM patients have stress adaptation disorders (11). This study showed that, after a 1-day outpatient intervention, stress hormones, including E and cortisol, were significantly reduced in GDM patients. At the same time, 2-h postprandial blood glucose and HOMA-IR decreased by 5.6 and 5.92%, respectively, indicating that 1-day outpatient intervention improved stress adaptation disorders in GDM patients and reduced 2-h postprandial blood glucose and insulin resistance levels, consistent with our previous research results (8).

In the GDM intervention group, E, cortisol, and the oxidative stress marker MDA were positively correlated with HOMA-IR, suggesting that insulin resistance in GDM patients may be closely related to stress adaptation disorders and oxidative stress. First, elevated levels of E and cortisol suggest that GDM patients may have overactivation of the HPA axis, which promotes hepatic gluconeogenesis and inhibits skeletal muscle glycogen synthesis, and promotes adipose tissue breakdown, leading to insulin resistance (12). Oxidative stress injury has been shown to be involved in the occurrence and progression of metabolic diseases (such as metabolic syndrome and diabetes) and is also associated with the processes of obesity and pregnancy (13). Elevated blood glucose further promotes the production of reactive oxygen species. In addition, increased electron supply to the mitochondrial respiratory chain and the activation of other metabolic pathways further increase in reactive oxygen species (14, 15). Increased oxidative stress causes abnormal insulin secretion and may even lead to the damage and apoptosis of pancreatic beta cells (16). In addition, oxidative stress can reduce the expression and content of glucose transporter 4 (GLUT4) in skeletal muscle, thereby contributing to insulin resistance by reducing cellular glucose uptake (17). Inflammatory reactions can also be enhanced by the body’s oxidative stress, which interferes with insulin signaling and leads to insulin resistance. GDM patients exhibit enhanced oxidative stress damage (18), and after 1-day outpatient intervention, the oxidative stress markers MDA and SOD in GDM patients were significantly reduced. The findings indicate that 1-day outpatient intervention exerts a beneficial effect on alleviating oxidative damage in GDM patients.

Iron overload also plays an important role in the occurrence and development of insulin resistance. Changes in iron levels can regulate insulin receptor phosphorylation levels, affecting the activity of key signaling molecules and subsequent signal transduction of insulin (19). In addition, iron overload could increase oxidative stress and inflammatory damage in the body, interfere with the transmission of insulin signaling pathways, and contribute to insulin resistance. In addition, the effects of iron overload may extend to transcription factors in glucose metabolism, iron response elements, and iron regulatory proteins, which jointly regulate iron homeostasis and the expression of genes related to energy metabolism, thereby affecting insulin sensitivity. Moreover, oxidative stress and inflammatory damage can also promote the occurrence of iron overload, and changes in iron levels can, in turn, affect the inflammatory response, forming a feedback loop that exacerbates insulin resistance in GDM patients under chronic inflammatory conditions (20, 21). Researchers have found that serum ferritin, serum iron, and transferrin saturation were significantly increased in GDM patients, indirectly suggesting that increased iron storage during pregnancy may be related to elevated blood glucose levels (22), consistent with our previous research results (23). At present, the complete intrinsic regulatory mechanism underlying the interaction among iron metabolism disorders, glucose homeostasis, and insulin resistance (HOMA-IR) has not been systematically elucidated. This study provides supplementary evidence on the association between iron metabolism, stress-related endocrine disorders, and glucose metabolism disorders at the population level, providing a reliable clinical basis for screening iron levels in target populations, early warning of gestational glucose metabolism abnormalities, and the development of personalized intervention strategies.

In this study, we found that following a 1-day outpatient intervention, serum ferritin (SF) levels in patients with GDM were significantly reduced. SF plays a central role in regulating iron metabolism; during inflammatory responses, various cytokines and inflammatory mediators can stimulate cellular ferritin synthesis, thereby elevating SF levels. Therefore, it not only functions as a key iron-storage protein but also acts as an acute-phase reactant in the context of inflammation (24). Multiple studies have reported that increased ferritin levels are associated with insulin resistance, although the underlying mechanisms remain incompletely elucidated (25–27). Our results have demonstrated that SF and iron (Fe) levels were positively correlated with malondialdehyde (MDA), a marker of oxidative stress injury, negatively correlated with superoxide dismutase (SOD), and positively correlated with HOMA-IR. These findings further support a close association between SF and the severity of oxidative stress injury and insulin resistance in patients with GDM.

Notably, the 1-day outpatient comprehensive intervention was multifaceted, including dietary prescription, exercise guidance, digital follow-up, and family education. After the intervention, both SF and MDA levels were significantly decreased, suggesting that the intervention alleviated systemic iron overload and oxidative stress injury, which may, in turn, contribute to improved insulin resistance. However, given the multi-component nature of the intervention, the independent contribution of iron metabolism modulation cannot be isolated, and the causal chain linking the intervention to changes in iron markers and subsequent improvements in insulin resistance remains speculative and requires further verification.

GDM patients often have adverse pregnancy outcomes. After the 1-day outpatient intervention, the weight gain of pregnant women in the GDM intervention group was significantly lower than that in the GDM group, and the insulin injection utilization rate of patients in the GDM intervention group was also significantly reduced (5.1%:16.9%). The incidence of postpartum blood glucose abnormalities was also significantly lower than that in the GDM group. In our study, the blood glucose control status of the two groups of GDM patients was evaluated through blood glucose testing results and self-monitoring records. If the blood glucose target could not be achieved within a 4–6-week window, insulin injection therapy was initiated. A study suggests that 95.3% of GDM patients could achieve their blood glucose goals through lifestyle interventions alone, and only 4.7% required medication treatment (28). This finding was consistent with the results of our study and further supports the effectiveness of 1-day outpatient interventions for GDM patients after diagnosis, including dietary and exercise habits. In addition, continuous excessive weight gain during pregnancy can lead to increased fat deposits in GDM women. As gestational weeks increase, the increase in fat mass may exacerbate insulin resistance and increase the use of insulin therapy (29). Our study found that 1-day outpatient intervention precisely controls the magnitude of weight gain in GDM pregnant women, which may be one of the important reasons for reducing insulin injection utilization.

We found that patients in the GDM intervention group showed a significant decrease in postpartum blood glucose, and the incidence of abnormal postpartum blood glucose was reduced. This finding was closely related to 1-day outpatient intervention, including core intervention measures such as standardized weight management during pregnancy, nutrition education, dietary guidance, and exercise guidance. At the same time, the decrease in incidence was directly related to regular outpatient follow-up during the intervention period, personalized nutrition guidance based on the WeChat platform, and health behavior patterns established during the intervention. Beyond clinical interventions, patients’ treatment compliance and self-management ability also govern the persistence of disease-specific behavioral patterns into the postpartum period, a factor that exerts a non-negligible impact on postpartum blood glucose homeostasis.

The incidences of cesarean delivery, preeclampsia, premature rupture of membrane in GDM intervention group were 6.4, 5.1, 3.8 and 2.6%, respectively. Body mass index and fetal birth weight at delivery were lower in GDM intervention group than those in the GDM group; however, the differences were not statistically significant. This may be due to the fact that fetal growth and development primarily occur after 24 weeks, and the screening time for GDM is relatively short compared to the delivery time, with only a 12–16-week interval having limited impact on the outcome of GDM patients (30).

One-day outpatient intervention for GDM patients covering diet treatment, exercise intervention, and healthy behavior shaping has been proven to be effective in controlling blood glucose, reducing stress adaptation disorder and oxidative stress injury. However, the intervention effect remains limited in improving cesarean section rate, pregnancy-induced hypertension syndrome incidence, preeclampsia incidence rate, risk of premature rupture of membranes, maternal body mass index at delivery, fetal birth weight, and other maternal and infant outcome indicators. In addition to the potential influencing factors mentioned earlier, the following aspects also need to be the focus of optimization in subsequent research: the sustainability of GDM patients’ compliance with intervention plans, whether the strong driving effect of insulin resistance on blood pressure and other outcomes interferes with intervention outcomes, limitations of single center study design, such as possible selection bias, and whether it affects the extrapolation validity of research conclusions.

Given the prevalence of GDM globally and its potential for adverse maternal and infant outcomes. One-day outpatient-based nutritional intervention ameliorates partial clinical outcomes among women with GDM, demonstrating promising potential for widespread clinical application. In addition, a 1-day outpatient intervention model can reduce insulin injection utilization, optimize the allocation of medical resources, and save costs. At the same time, by regulating the rate of weight gain during pregnancy and promoting healthy eating habits, it can reduce the risk of recurrence of GDM and the risk of transformation into type 2 diabetes and has a significant impact on the development of their own eating habits and the risk of chronic metabolic diseases such as diabetes, hypertension, and hyperlipidemia in adulthood. The potential benefits mentioned above need to be further validated through well-designed, adequately sampled, and long-term follow-up randomized controlled trials, providing high-level evidence to support improvements in GDM pregnancy management plans.

## Data Availability

The raw data supporting the conclusions of this article will be made available by the authors, without undue reservation.
